# Plastome‐Based Phylogenomics Resolves Phylogenetic Complexity in *Lysimachia* and Reveals Unexpected Placement of *L. thyrsiflora*

**DOI:** 10.1002/ece3.74544

**Published:** 2026-11-04

**Authors:** SeongJun Park, JeongYun Byeon, SeonJoo Park

**Affiliations:** ^1^ Department of Life Sciences Yeungnam University Gyeongsan Gyeongsangbuk‐do Republic of Korea

## Abstract

The genus *Lysimachia* is taxonomically complex, especially in East Asia. We analyzed the complete chloroplast (cp) genomes of four *Lysimachia* species (*L*. *acroadenia*, *L. leucantha* , *L. thyrsiflora* , and *L. arvensis* ). The cp genomes (153–155 kb) showed the typical quadripartite structure. Comparative analysis identified two highly variable regions, which are promising molecular markers. Most protein‐coding genes showed Ka/Ks ratios below 1, but *rps*12 and *psa*I showed elevated Ka/Ks ratios that require further validation using codon‐based selection tests. The Maximum likelihood phylogenetic tree confirmed the monophyly of *Lysimachia*. Importantly, *L. thyrsiflora* clustered within subgenus *Lysimachia* section *Lysimachia*, contradicting its traditional classification and suggesting a complex biogeographic history, potentially involving historical connections across the Bering Land Bridge. Divergence time analysis estimated the genus origin at approximately 33 Mya. Our results clarify the phylogenetic status of key taxa and provide essential genomic resources for future evolutionary and taxonomic research on *Lysimachia*.

## Graphical abstract

We investigated the phylogenetic relationships and evolutionary history of Lysimachia using complete chloroplast genomes from four species. Comparative and phylogenetic analyses revealed conserved genome structure, identified variable regions as potential molecular markers, and detected positive selection in specific genes. Our results support the monophyly of the genus, revise the placement of *L. thyrsiflora* , and provide insights into its evolutionary history and divergence timing.

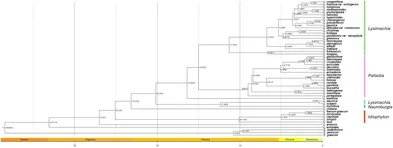

## 1 Introduction

According to the APG IV classification system, the genus *Lysimachia* Tourn. ex L. is classified under the family Primulaceae and the subfamily Myrsinoideae (Angiosperm Phylogeny Group et al. 2016). The genus *Lysimachia* is known to contain about 280 species distributed worldwide, according to Kew's Plants of the World Online (POWO) database (Govaerts et al. 2021). East Asia (Korea, China, and Japan) shows a higher diversity than similar latitude areas in North America or Europe, with about 170 species of *Lysimachia* distributed in East Asia (Yan et al. 2018). It is also one of the genera in which new species are still being announced and research is being conducted (Liu et al. 2014; Zhou et al. 2015; Yan et al. 2017; Ju et al. 2021; Tae et al. 2010). The genus *Lysimachia* was traditionally included in the tribe Lysimachieae together with *Trientalis* L., *Glaux* L., *Anagallis* L., *Asterolinon* Hoffmanns. & Link, and *Pelletiera* A. St.‐Hil. (Anderberg et al. 2007). However, these genera have now been integrated into the genus *Lysimachia* by molecular analyses such as Internal Transcribed Spacer (ITS) (Manns and Anderberg 2009).

The genus *Lysimachia* has been the subject of attempts by various scholars to define its subgenus boundaries since earlier times. F.W. Klatt, an early researcher, divided it into nine sections based on morphological similarities such as flower structure and inflorescence (Klatt 1866). Afterwards, R. Knuth divided the genus *Lysimachia* into 16 sections using ecological and morphological characteristics (Pax and Knuth 1905). Handel‐Mazzetti later classified the genus into five subgenera based on floral structure and stamen development. He reorganized the genus to reflect the evolutionary relationships of the flowers, which clarified the boundaries between the subgenera (Handel‐Mazzetti 1928). Most recently, Chen & Hu revised the Handel‐Mazzetti system, dividing the genus into six subgenera: *Idiophyton*, *Lysimachia*, *Palladia*, *Heterostylandra*, *Naumburgia*, and *Lysimachiopsis* (Feng‐Hwai and Chi‐Ming 1979).

Molecular studies have supported the monophyly of *Lysimachia* but have also identified polytomies and uncertain relationships among several taxa (Hao et al. 2004; Anderberg et al. 2007). Complete chloroplast genomes can complement nuclear markers by providing data for phylogenetic reconstruction, comparison of genome structure, identification of variable regions for molecular markers, and evaluation of closely related or morphologically similar taxa. However, discrepancies between chloroplast‐based and nuclear phylogenies indicate that broader plastome sampling is needed to clarify infrageneric relationships and species placement within *Lysimachia* (Liu et al. 2023).

Currently, 16 taxa of *Lysimachia* plants are known to exist in Korea. In this study, we investigated four species that are distributed in specific environments, such as wetlands and islands. First, *Lysimachia acroadenia* Maxim. is native to both Korea and Japan, with its distribution in Korea limited to Jeollanam‐do and Jeju‐do. This species typically inhabits moist areas within forests (National Institute of Biological Resources 2021). Second, *Lysimachia leucantha* Miq. is also native to Korea and Japan. In Korea, it is found in wetlands across Gyeongsangnam‐do, Jeollanam‐do, and Jeju Island. Morphologically similar to *L. acroadenia*, *L. leucantha* can be distinguished by its dense racemose inflorescences. Third, *Lysimachia thyrsiflora* L. is distributed across subarctic regions of the Northern Hemisphere. In Korea, its occurrence is highly restricted, being found only in a single wetland in Gangwon‐do. Finally, *Lysimachia arvensis* (L.) U. Manns & Anderb. is native to Europe, Africa, and the Mediterranean region (Manns and Anderberg 2007). In Korea, it has been reported only from Jeju Island. This species is characterized by its circumscissile capsule and a blue or orange corolla divided into five lobes. Recently, there is a study related to flower color polymorphism, which seems to be still controversial (Manns and Anderberg 2005; Sánchez‐Cabrera et al. 2024). According to Chen and Hu, *L. thyrsiflora* is characterized by small, round stamen heads and is classified within the subgenus *Naumburgia* due to its adaptation to wetland habitats. The remaining three species were not included from the classification by Chen and Hu. This is because *L. acroadenia* and *L. leucantha* are known to be native only to Korea and Japan (National Institute of Biological Resources 2021; Ōi 1965), while *L. arvensis* was previously classified under the genus *Anagallis* (Manns and Anderberg 2009).

The chloroplast genome is a useful resource for plant phylogenomic and comparative genomic studies because its generally conserved structure is combined with lineage‐specific variation in nucleotide sequences, repeat regions, and inverted‐repeat boundaries (Kim, Ha, et al. 2022). The chloroplast (cp) genome has a circular quadripartite structure consisting of a large single‐copy (LSC) region, a small single‐copy (SSC) region, and two inverted repeat (IR) regions. The size of the cp genome ranges from 120 to 180 kb, and 110–130 genes are encoded (Duan et al. 2020; Choi et al. 2015; Jiang et al. 2023). The cp genome has uniparental inheritance, mostly maternal, and low genetic variation. Also, the cp genome has a stable structure, multiple copies, and conserved sequences. These characteristics play an important role in phylogenetic and evolutionary studies (Park and Son 2024; Huang et al. 2023; Ran et al. 2024). In *Lysimachia*, these features can be used to reconstruct phylogenetic relationships, identify candidate molecular markers for species discrimination, compare genome structure across taxa, and examine patterns of nucleotide substitution.

We sequenced and analyzed the complete chloroplast genomes of four *Lysimachia* species native to wetland and island habitats in Korea. Among them, *L. thyrsiflora* was previously classified under subgenus *Naumburgia*. In this study, we analyzed the chloroplast genome of *L. thyrsiflora* as a representative of subgenus *Naumburgia* for the first time. The objectives of this study were as follows: first, to characterize chloroplast genome structure and sequence variation among the studied *Lysimachia* taxa; second, to identify variable regions that may serve as candidate molecular markers and to examine nucleotide substitution patterns; and finally, to evaluate whether complete chloroplast genome data can clarify phylogenetic relationships and the placement of taxa with uncertain classification within *Lysimachia*.

## 2 Materials and Methods

### 2.1 Genomic DNA Isolation and DNA Sequencing

Fresh leaves of four species of *Lysimachia* (*L*. *acroadenia*, *L. leucantha* , *L. thyrsiflora* , *and L. arvensis*) were collected from their natural habitats. Genomic DNA was isolated from the leaves using the Exgene Plant SV Kit (GeneAll Biotechnology Inc., Seoul, Republic of Korea). The quantity and purity of gDNA were measured using a Nano‐200 spectrophotometer (Hangzhou Allsheng Instruments Inc., Hangzhou, China) and assessed using 1% agarose gel electrophoresis. NGS was performed using Illumina HiSeq2500 platform (Phyzen Ltd., Seongnam, Republic of Korea). Paired‐end sequencing with an insert size of 550 bp was conducted, yielding approximately 3 Gb of raw data for each of the forward and reverse reads.

### 2.2 Assembly and Annotation of Four *Lysimachia* Chloroplast Genomes

De novo assembly of the raw sequencing data was performed using Velvet v1.2.10 (Zerbino and Birney 2008). The assembled cp genomes were then assessed for coverage using reference assembly in Geneious Prime v2025.1.2 (Biomatters Ltd., Auckland, New Zealand). For cp genome annotation, the GeSeq tool and NCBI BLASTN searches were used, and visualization was conducted using OGDRAW (Lohse et al. 2007; Tillich et al. 2017).

### 2.3 Comparative Analysis of Chloroplast Genomes Structure and Genetic Variation

The four *Lysimachia* chloroplast genomes were analyzed in comparison with 45 previously published *Lysimachia* cp genomes (Table S1). In total, 49 complete cp genomes were used to compare sequence similarity using the shuffle‐LAGAN mode in mVISTA, with *L. europaea* , the basal species, used as the reference (Frazer et al. 2004). For the IR boundary analysis, the junctions between the LSC, SSC, and IR junctions were examined to investigate the expansion and contraction of the cp genomes (Amiryousefi et al. 2018).

To compare the genetic variation within the genus *Lysimachia*, 49 cp genomes were aligned using MAFFT implemented in Geneious Prime (Biomatters Ltd., Auckland, New Zealand) (Katoh and Standley 2013). Subsequently, nucleotide diversity (Pi) was calculated using DnaSP v6.12.03 to identify highly variable regions. The analysis was performed with a window length of 800 bp and a step size of 200 bp (Rozas et al. 2017).

### 2.4 Repeat Sequence Analysis

Repeat sequence analysis was conducted using 49 cp genomes, focusing on the LSC, SSC, and one of the IR regions. For the analysis of simple sequence repeats (SSRs), MISA‐web v2.1 was used with the following parameters: a minimum of 10 repeat units for mononucleotide SSRs, 6 repeat units for dinucleotide SSRs, and 5 repeat units for tri‐, tetra‐, penta‐, and hexanucleotide SSRs (Beier et al. 2017). Tandem repeats were identified using Phobos Tandem Repeat Finder v1.0.6, with the parameters set as follows: 1 for matches, −5 for mismatches and gaps, and 0 for N positions (Phobos 2010). Forward reverse, complement, and palindromic repeats were analyzed using REPuter, with the parameters set to a Hamming distance of 3, a minimum sequence identity of 90%, and a minimum repeat size of 30 bp (Kurtz et al. 2001).

### 2.5 Relative Synonymous Codon Usage

Codon usage bias in the genus *Lysimachia* was analyzed using DnaSP v6.12.03 based on 76 shared protein‐coding genes, including stop codon (Rozas et al. 2017). Relative synonymous codon usage (RSCU) values were calculated, and the results were visualized as a heat map using the R package ComplexHeatmap (Gu et al. 2016).

### 2.6 Substitution Estimates

The substitution rates within the genus *Lysimachia* were estimated using 49 complete chloroplast genomes. A total of 76 shared functional protein‐coding genes were extracted from the chloroplast genomes and treated as orthologous gene sets. For each gene, coding sequences were aligned using the Translation Align function in Geneious Prime (Biomatters Ltd., Auckland, New Zealand), and stop codons were removed before analysis. Pairwise sequence comparisons were then performed among the 49 *Lysimachia* taxa for each orthologous gene. Nonsynonymous (Ka) and synonymous (Ks) substitution rates, as well as Ka/Ks ratios, were calculated for each pairwise comparison using DnaSP v6.12.03 (Rozas et al. 2017). The mean Ka, Ks, and Ka/Ks values for each gene were calculated from all pairwise comparisons and used for subsequent analyses.

### 2.7 Phylogenetic and Divergence Time Analysis

A phylogenetic tree was constructed using 49 *Lysimachia* cp genomes and three outgroup cp genomes. The 45 *Lysimachia* cp genomes and three outgroup cp genomes were downloaded from public databases. The phylogenetic analysis was based on 76 protein‐coding genes shared across all 52 cp genomes (Table S1). Sequence alignment was performed using MAFFT v1.5.0, and the aligned sequences were saved in FASTA format (Katoh and Standley 2013). A maximum likelihood (ML) tree was generated using IQ‐TREE v2.4.0 with the GTR + F + R4 model, which was selected based on the Bayesian information criterion (BIC), and branch support was assessed with 1000 bootstrap replicates (Minh et al. 2020).

Divergence analysis of *Lysimachia* was performed using Bayesian inference with Bayesian Markov Chain Monte Carlo (MCMC) sampling implemented in BEAST v1.10.4 (Suchard et al. 2018). An uncorrelated relaxed clock with a lognormal distribution model was applied, and the MCMC analysis was run for 300 million steps. Two calibration priors were used, including a fossil‐based calibration and a secondary calibration. The first calibration point was placed at the most recent common ancestor (MRCA) of *L*. *davurica*, *L. thyrsiflora* , and *L. vulgaris* at 12.45 million years ago (Mya) (12.06–15.6 Mya, lognormal distribution) (Friis 1985), based on Middle Miocene fossil seeds similar to *L. vulgaris* described by Friis. This calibration was assigned a lognormal prior with an offset of 12.0 Mya, a mean of 1.0 and a standard deviation of 2.0. The second calibration point was set for the MRCA of all *Lysimachia* species except *L. europaea* at 28.5 Mya (21.92–35.08 Mya, normal distribution), following the secondary calibration estimate of Yan et al. (Yan et al. 2018). This calibration was assigned a normal prior with a mean of 28.5 Mya and a standard deviation of 4.0 Mya. All effective sample size (ESS) values were confirmed to exceed 200 using Tracer v1.7.2 (Rambaut et al. 2018). TreeAnnotator v1.10.4 was also used with a burn‐in of 10% (Suchard et al. 2018).

## 3 Results

### 3.1 General Features of the Four *Lysimachia* Chloroplast Genome

The four *Lysimachia* cp genome sequences ranged from 153,325 to 155,181 bp in length and were composed of a LSC, a SSC, and two inverted repeats (IRa/IRb) (Figure 1). The cp genome comprised a total of 113 unique genes, including 85 protein‐coding genes, 30 tRNA genes, and 4 rRNA genes. Due to duplication by the IR regions, an additional 6 protein‐coding genes, 7 tRNA genes, and 4 rRNA genes are duplicated, resulting in a total of 130 genes present in the genome (Table S2). The GC content of the four cp genomes ranged from 36.8% to 37.0%, with the highest GC content observed in the IR regions, followed by the LSC and SSC regions (Table 1). In *L. thyrsiflora* , the highest GC content was found in the tRNA genes, whereas in the remaining species, the highest GC content was observed in the rRNA genes.

**FIGURE 1:**
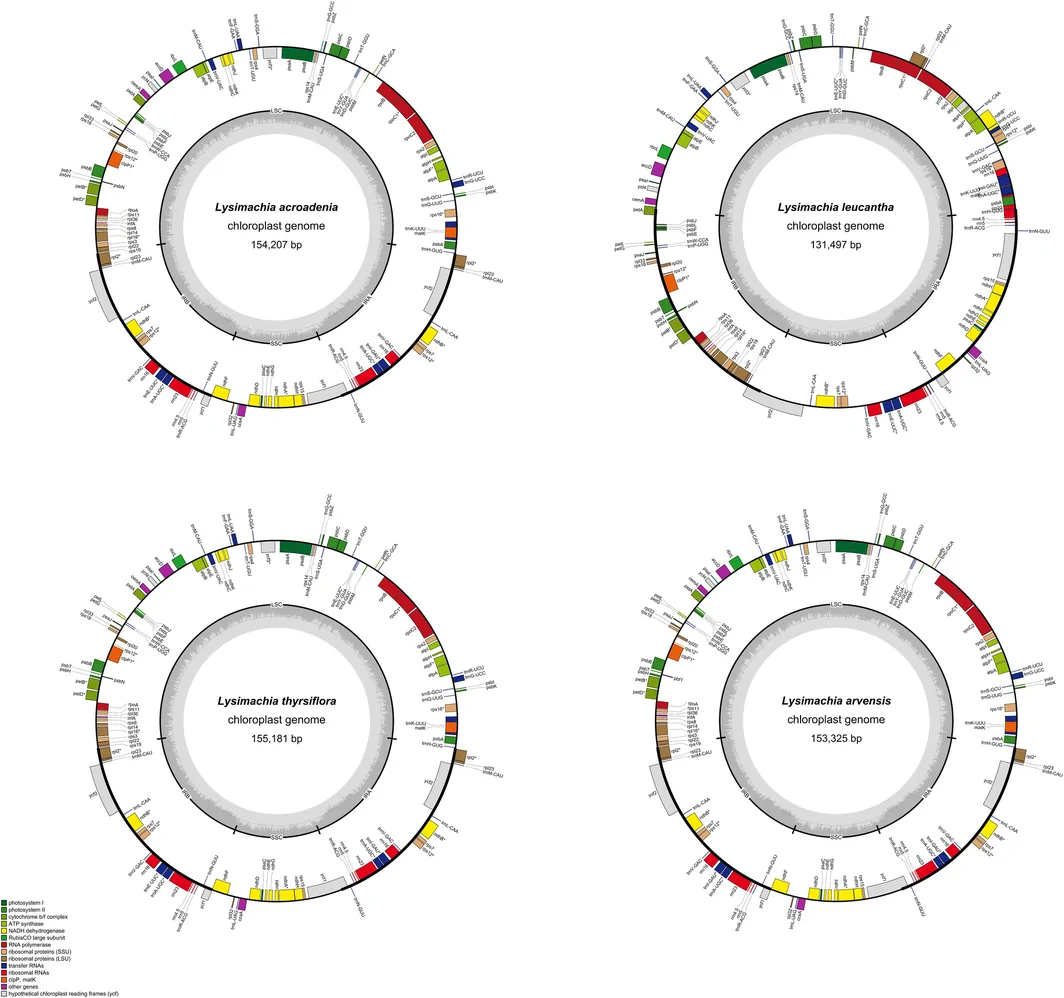
Circular chloroplast (cp) genome map of 4 *Lysimachia*. Genes shown outside the large circle are transcribed clockwise, and those inside the circle are transcribed counterclockwise. Genes are color‐coded according to their different functional groups. The large single‐copy (LSC), small single‐copy (SSC), and two inverted‐repeat (IRa and IRb) regions are indicated on the maps. The gray inner circle represents GC content across the cp genomes.

**TABLE 1:** Characteristic features of the 4 *Lysimachia* chloroplast genome.

Sequence region	*L*. *acroadenia*	*L. leucantha*	*L. thyrsiflora*	*L. arvensis*
*Genome size (bp)*	154,207	153,659	155,181	153,325
LSC	84,300	83,692	85,031	83,828
SSC	17,981	17,949	18,044	18,045
IR	25,963	26,009	26,053	25,726
*GC content* (%)				
Total	37.0	37.0	37.0	36.8
LSC	35.0	34.9	34.9	34.6
SSC	30.3	30.4	30.3	30.0
IR	42.8	42.7	42.8	42.8
CDS	37.3	37.2	37.3	37.1
tRNA	53.0	53.0	55.3	52.8
rRNA	55.3	55.2	52.9	55.3

### 3.2 Comparative Analysis of Sequence Divergence in the *Lysimachia* cp Genomes

The structural analysis using mVISTA revealed that the taxa were divided into two major groups (Figure S1). The first group, representing the basal lineages, included subg. *Naumburgia*, subg. *Lysimachia* sect. *Lysimachia*, subg. *Idiophyton*, *Anagallis* clade, and *Trientalis* clade. The second group comprised the remaining taxa. In the second group, two notable deletions were identified in the intergenic spacer (IGS) regions: approximately 600 bp in the *pet*N‐*psb*M region and about 450 bp in the *ycf*3‐*rps*4 region. In addition, subg. *Idiophyton* also showed low sequence similarity in the IGS region between *trn*E‐*trn*T. Overall, the IR regions were found to be highly conserved. However, some taxa, such as *L. heterogenea* and *L. mauritiana* , exhibited low sequence similarity in the IGS region between *rps*7 and *trn*V.

The IR and SC boundaries were compared among 49 *Lysimachia* species (Figure S2). *L*. *chekiangensis* was found to have the longest LSC region (85,181 bp), while *L. stenosepala* had the longest SSC region (18,203 bp). In the case of the IR region, *L. capillipes* exhibited the greatest length (26,258 bp). In most taxa, the gene *rps*19 was located at the boundary between the LSC and IRb regions. However, in some species of subg. *Palladia* sect. *Miltandrae*, *rps*19 was entirely located within the LSC region. In *subg. Idiophyton*, the majority of the *rps*19 gene was positioned within the IRb region. At the boundary between the SSC and IRa regions, *ycf*1 was present in most taxa; however, in *L. stenosepala*, *ycf*1 existed as a pseudogene within the SSC region.

To examine nucleotide diversity within the genus *Lysimachia*, Pi values were calculated using 49 cp genomes (Figure 2). The average nucleotide diversity was 0.013, and the highly conserved IR regions exhibited low diversity (Pi < 0.01). The highest Pi value (0.052) was observed in the *ycf*1 region located in the SSC. The second highest Pi value was observed in the *ndh*F‐*rpl*32 region within the SSC (0.038). Other highly variable regions included *trn*K‐*rps*16 (0.035), *rpl*22 (0.035), and *rpo*B‐*psb*M (0.035) in the LSC region.

**FIGURE 2:**
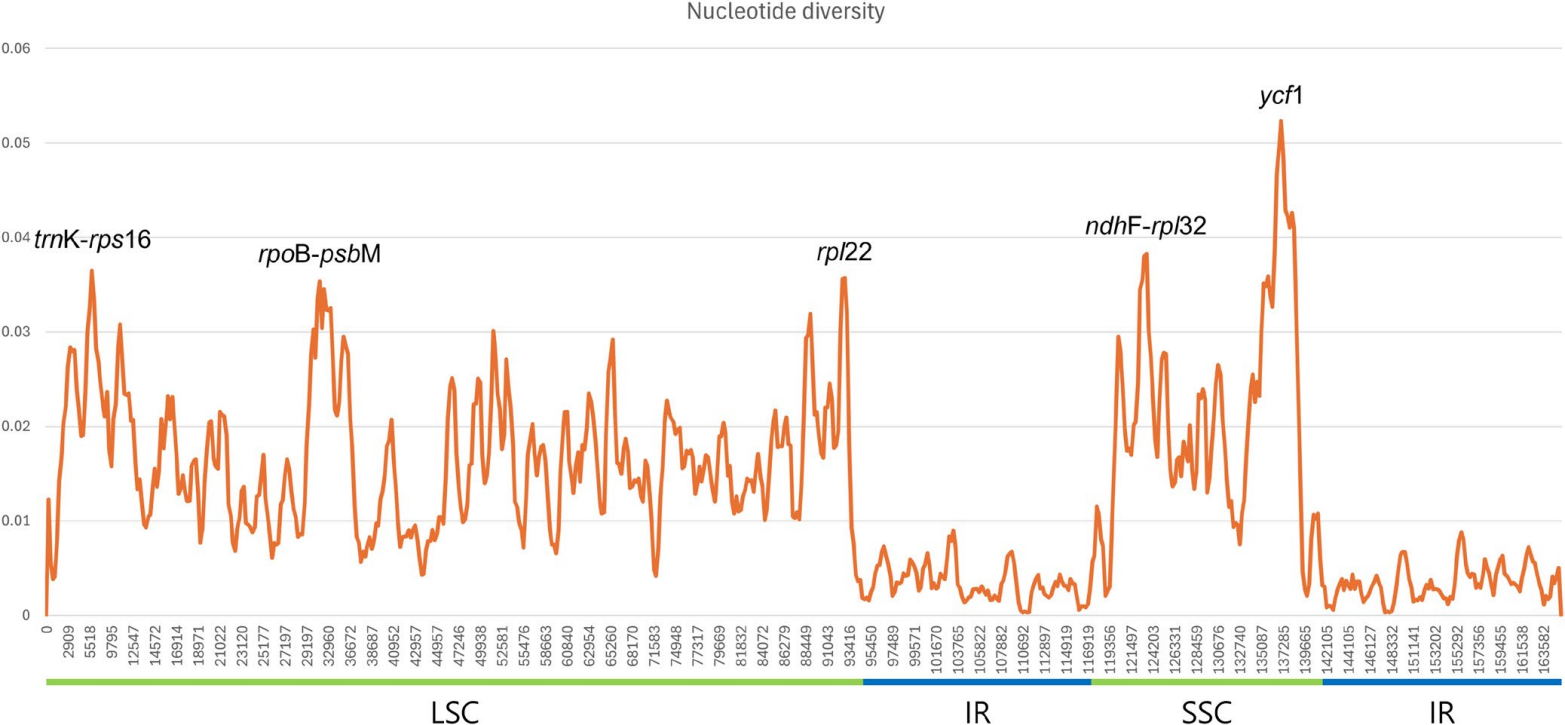
Sliding window analysis of the whole cp genome showing nucleotide diversity (Pi) compared across 49 *Lysimachia* species.

### 3.3 Comparative Analysis of the Repeat Sequences in the *Lysimachia* cp Genomes

The SSR analysis revealed a range of 37 (in *L. coreana* ) to 68 (in *L*. *alfredii*) SSRs among the genus (Figure 3A). Four types of SSRs were identified within the genus *Lysimachia*, with mononucleotide SSRs present in all taxa. Dinucleotide SSRs were absent in five species, while trinucleotide SSRs were detected in 19 species. Tetranucleotide SSR was found only in *L. arvensis* . A total of 2250 SSRs were identified, of which mononucleotide repeats accounted for 95.2%, di‐ for 3.7%, tri‐ for 0.9%, and tetra‐ for 0.04%.

**FIGURE 3:**
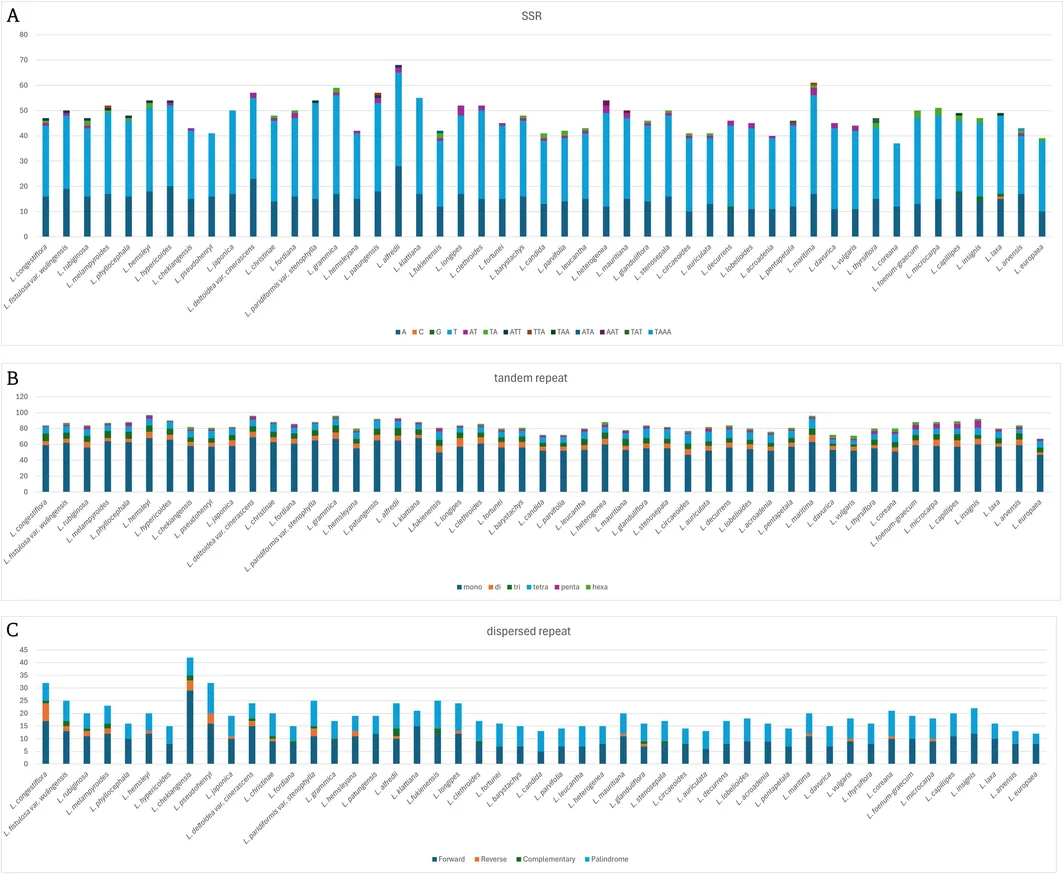
A: Distribution of SSRs in *Lysimachia*, B: Distribution of tandem repeats in *Lysimachia*, C: Distribution of dispersed repeats in *Lysimachia*. The x‐axis represents the examined *Lysimachia* species, and the y‐axis represents the number of repeats.

The number of tandem repeats ranges from 37 (in *L. europaea* ) to 68 (in *L*. *hemsleyi*) (Figure 3B). Mononucleotide repeats were the most abundant in all species. The second most frequent repeat type varied among taxa, appearing as di‐, tri‐, or tetra‐nucleotide repeats depending on the species. Hexanucleotide repeats were not detected in *L. europaea* alone. A total of 4104 tandem repeats were identified, consisting of 69.15% mono‐, 7.29% di‐, 8.31% tri‐, 9.82% tetra, 3.36% penta‐, and 2.07% hexanucleotide repeats.

The number of dispersed repeats ranged from 12 (in *L. europaea* ) to 42 (in *L*. *chekiangensis*) (Figure 3C). Among all species, forward repeats were the most abundant, followed by palindromic repeats. Reverse and complementary repeats occurred at similar frequencies. A total of 937 dispersed repeats were identified, comprising 53% forward, 4.2% reverse, 2.2% complementary, and 40.6% palindromic repeats.

The total number of repeats ranged from 118 in *L. europaea* to 185 in *L*. *alfredii* (Table S3).

### 3.4 RSCU in the *Lysimachia* cp Genomes

The RSCU analysis revealed that codon usage patterns were highly conserved across all *Lysimachia* species (Figure 4). Codons with high RSCU values included UUA (Leu), GCU (Ala), UCU (Ser), and ACU (Thr), whereas codons such as CUG (Leu), AGC (Ser), and UAC (Tyr) exhibited low RSCU values. In addition, the third position of codons was predominantly occupied by A or U, indicating an overall AT‐rich codon bias in the chloroplast genome of *Lysimachia*.

**FIGURE 4:**
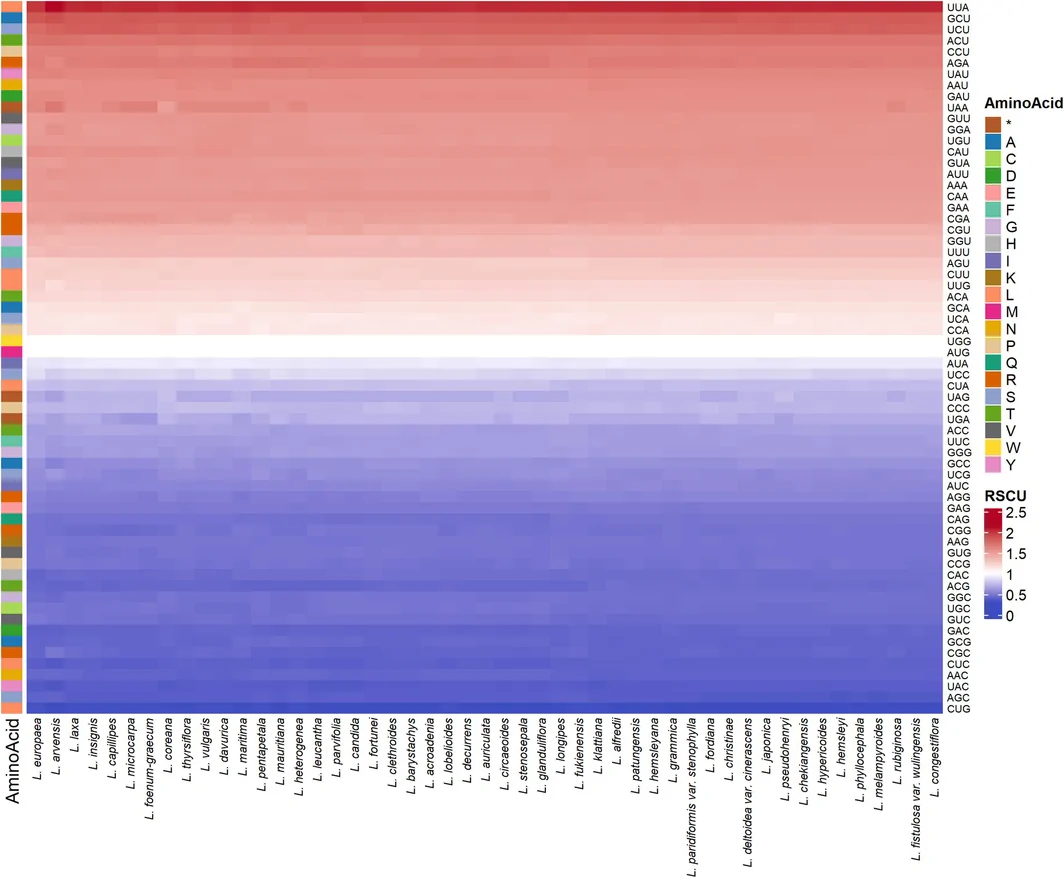
Heatmap showing the relative synonymous codon usage (RSCU) across 49 *Lysimachia* species. Amino acids are indicated by colored boxes on the left, and the corresponding amino acid names for each color box are listed on the right.

### 3.5 Nonsynonymous and Synonymous Substitution Estimates in the *Lysimachia* cp Genomes

The Ka and Ks values were calculated using 76 shared protein‐coding genes from 49 *Lysimachia* species (Figure 5; Table S4). Ka values were below 0.02 for most genes, whereas *rpl22* and *rpl32* showed Ka values exceeding 0.02. Similarly, Ks values were below 0.05 for most genes; however, eight genes (*ndhF*, *rps8*, *petD*, *rpl22*, *psaJ*, *psbK*, *rpl14*, and *rps19*) exhibited Ks values greater than 0.05. Ka/Ks ratios were below 1 for most genes. In contrast, *rps12* (1.21) and *psaI* (1.17) exhibited Ka/Ks ratios greater than 1.

**FIGURE 5:**
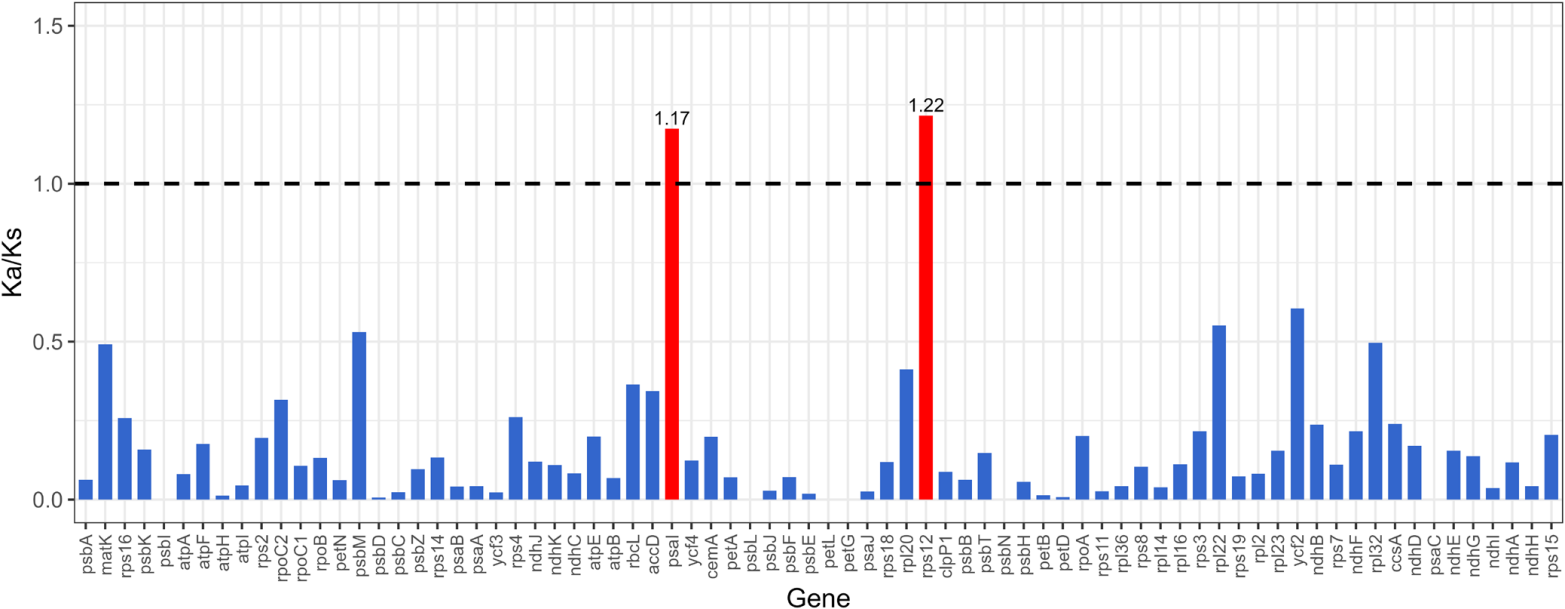
Selective pressure of 76 protein‐coding genes across the 49 *Lysimachia*.

### 3.6 Phylogenetic Analysis of the Genus *Lysimachia*

Using 76 shared protein‐coding genes from 49 *Lysimachia* species and 3 outgroups, a maximum likelihood (ML) tree was constructed (Figure 6). *Lysimachia* formed a monophyletic group. While most subgenera were monophyletic, several sections did not form monophyletic groups. *L. thyrsiflora* was included in subgenus *Lysimachia* section *Lysimachia* and clustered with *L*. *davurica* and *L. vulgaris* . Additionally, *L. arvensis* , formerly classified in *Anagallis*, was found to form a long branch. *L*. *acroadenia* was placed at the basal position of subgenus *Palladia* and showed a close phylogenetic relationship with *Lysimachia* species distributed in China and Japan. In addition, *L. leucantha* was located in the upper part of subgenus *Palladia*, and both species exhibited high bootstrap support values.

**FIGURE 6:**
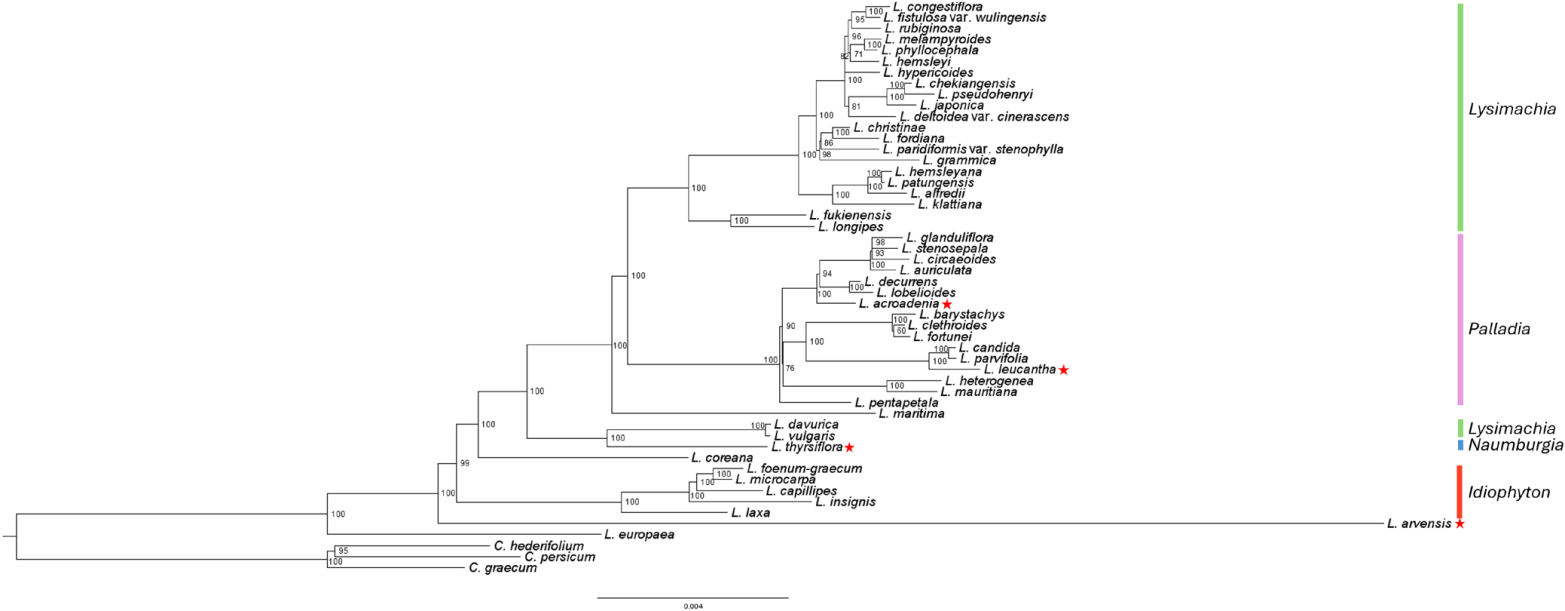
Maximum likelihood (ML) tree for 52 taxa based on 76 plastid protein‐coding genes. Values above the branches represent the ML bootstrap value. Red stars indicate the plastid genomes newly generated in this study.

The divergence time analysis indicated that the genus *Lysimachia* diverged in the early Oligocene, approximately 33 million years ago (95% HPD: 24.47–42.99 Mya) (Figure 7, Figure S3). Most subgenera were found to have diverged since the middle Miocene, around 15 Mya or earlier. Many lineages appear to have diverged more recently, particularly within the subgenera *Lysimachia* and *Palladia*.

**FIGURE 7:**
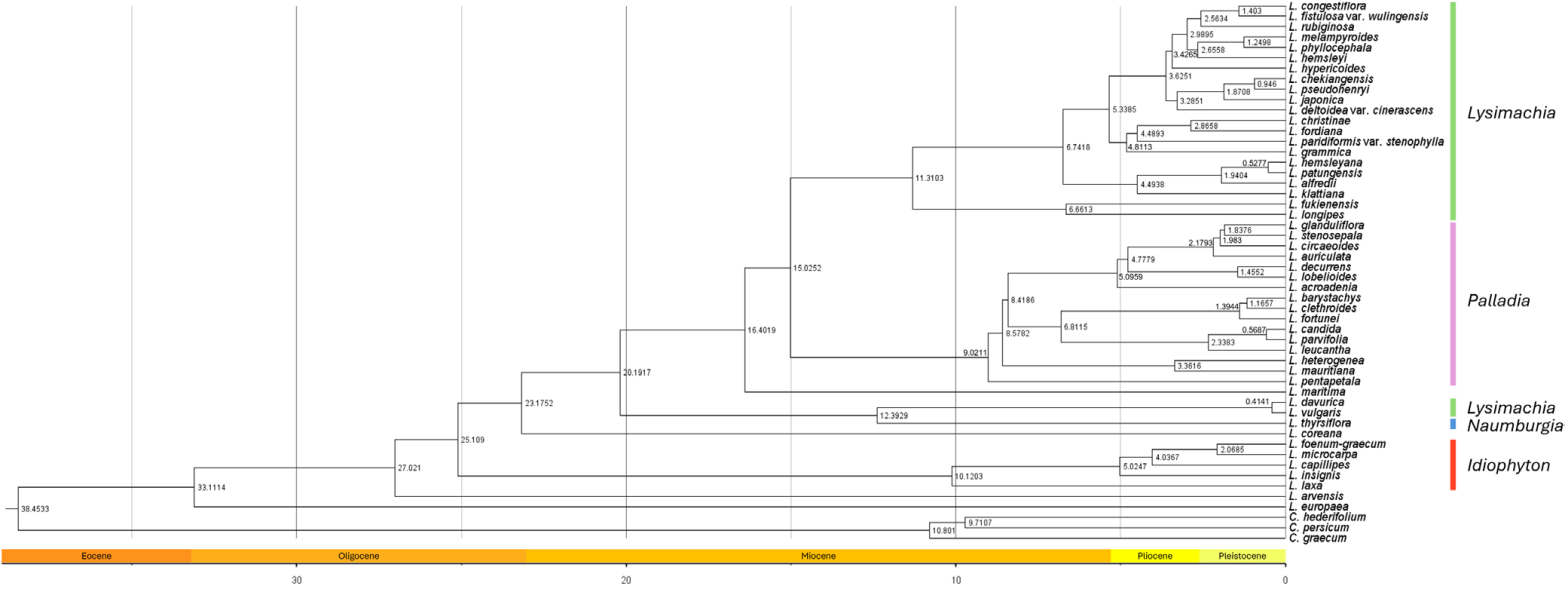
Divergence time estimation of *Lysimachia* species based on 76 chloroplast protein‐coding genes from 49 *Lysimachia* species and three outgroup taxa.

## 4 Discussion

### 4.1 Structural Variation and IR Boundary Dynamics in *Lysimachia*

Comparative analysis of the *Lysimachia* cp genomes revealed that their overall genomic structures are highly conserved, a pattern consistent with most angiosperms (Kim et al. 2025; Lee et al. 2006; Raman and Park 2016). The mVISTA analysis showed that sequence divergence was mainly concentrated in the SC regions, while the IR regions were remarkably conserved across taxa. This structural stability of the IR regions is considered a key factor maintaining the overall integrity of cp genomes, as the duplicated nature of IRs suppresses large‐scale rearrangements and enhances genomic stability (Figure S1) (Yue et al. 2008).

Despite the general conservation, several lineage‐specific variations were detected. Two notable deletions were found in the *pet*N‐*psb*M and *ycf*3‐*rps*4 intergenic spacers in a particular clade of taxa (Figure S1). These variants may represent synapomorphic characters defining specific lineages or clades. Moreover, the comparative analysis of IR and SC boundaries among 49 *Lysimachia* species revealed subtle but significant boundary shifts (Figure S2). In most taxa, the *rps*19 gene was positioned at the LSC/IRb junction. However, in some species belonging to subg. *Palladia* sect. *Miltandrae*, *rps*19 was entirely located within the LSC region, indicating an IR contraction. In contrast, in subg. *Idiophyton*, most of rps19 extended into the IRb region, suggesting an IR expansion. At the SSC/IRa junction, the *ycf*1 gene was generally found at the boundary, but in *L*. *stenosepala* it appeared as a pseudogene within the SSC, implying a lineage‐specific rearrangement or partial gene loss.

These patterns of IR expansion and contraction are common mechanisms contributing to chloroplast genome size variation among angiosperms (Cauz‐Santos 2025). Their occurrence within *Lysimachia* indicates that, although the genus largely maintains a conserved chloroplast genome architecture, it has experienced localized structural evolution during diversification. Such boundary dynamics may reflect both random events and lineage‐specific selective constraints acting on the plastid genome organization. Overall, these structural variations offer potential genomic markers for resolving subgeneric relationships within the genus (Park et al. 2021).

Beyond structural variation, codon usage patterns were also highly conserved across the genus (Figure 4). RSCU analysis revealed a preference for A/U‐ending codons (such as UUA, GCU, and UCU), indicating an AT‐rich codon bias typical of angiosperm plastomes (Zhang and Feng 2025; Rao et al. 2024). The conserved codon usage pattern among species indicates functional constraints on chloroplast gene expression and translation efficiency (Suzuki and Morton 2016).

Ka/Ks analysis further supported this pattern of strong evolutionary constraint (Figure 5). Most protein‐coding genes showed Ka/Ks < 1, consistent with purifying selection. However, two genes, *rps*12 and *psa*I, displayed elevated Ka/Ks ratios greater than 1. Because these values were estimated from plastome‐derived substitution rates alone, they should be regarded as preliminary signals requiring confirmation using codon‐based selection tests and additional genomic evidence. In addition, *rps*12 exhibited high substitution rates in exon 1 located in the LSC region, consistent with previous studies (Ping et al. 2021).

### 4.2 Genomic Hotspots for Molecular Markers

The distribution of nucleotide diversity and variation in repeat sequences observed in the chloroplast genomes provide valuable indicators for species delimitation and identification (Zhang et al. 2023). In particular, the *ycf*1 and *ndh*F‐*rpl*32 regions exhibited high Pi values, suggesting their potential to resolve phylogenetic relationships among closely related species (Figure 2). Repeat sequences also showed substantial interspecific variation in both number and composition (Figure 3). Species‐specific repeat patterns, when combined with nucleotide diversity, can thus serve as powerful diagnostic characters for distinguishing taxa.

Overall, the combination of highly variable genomic regions and repeat sequence diversity is expected to be especially useful for identifying morphologically similar taxa and can be applied to practical fields such as plant quarantine and authentication of medicinal plant materials (Lee et al. 2019; Kim, Oh, et al. 2022). As a result, they hold significant promise for increasing taxonomic resolution, ensuring the accuracy of species‐level identifications, and supporting conservation and resource‐management strategies within *Lysimachia*.

These repeat and nucleotide‐variation patterns provide complementary insights into plastome evolution among the examined *Lysimachia* taxa (Liu et al. 2023). Interspecific variation in SSRs, tandem repeats, and dispersed repeats may reflect lineage‐specific sequence dynamics and identify candidate regions for chloroplast marker development (Liu et al. 2023; Zhang et al. 2023; Lee et al. 2019; Kim, Oh, et al. 2022). In contrast, the broadly similar RSCU patterns suggest that codon usage is largely shaped by conserved nucleotide composition and functional constraints on plastid protein‐coding genes, rather than by pronounced lineage‐specific divergence (Suzuki and Morton 2016). Together with the variable regions identified by nucleotide‐diversity analysis, these features provide a useful genomic framework for evaluating relationships among closely related taxa and for selecting candidate plastid markers (Liu et al. 2023; Zhang et al. 2023). However, their utility for species delimitation requires validation using broader intraspecific and interspecific sampling, together with independent nuclear markers and morphological evidence (69).

### 4.3 Phylogenetic Insights Into the Evolution and Taxonomic Revision of *Lysimachia*

Using 76 shared protein‐coding genes, we reconstructed a phylogeny for 49 *Lysimachia* taxa (Figure 6). *Lysimachia* formed a well‐supported monophyletic group with strong bootstrap values, consistent with previous phylogenetic studies of the genus. Most subgenera were recovered as monophyletic. However, subgenus *Lysimachia* was divided into two clades corresponding to section *Lysimachia* and section *Nummularia*. Several sections were recovered as non‐monophyletic groups, suggesting that traditionally used morphological characters do not reflect the evolutionary signals detected by the chloroplast genome.

The structural characteristics of the chloroplast genomes and the phylogenetic classification within the genus *Lysimachia* were largely congruent at the subgeneric level (Figures S1 and S2). The mVISTA results indicated that two indels were detected exclusively in *L. maritima* and the upper clade, while another indel was specific to subgenus *Idiophyton*. In addition, IR boundary analysis revealed a translocation of rps19 caused by IR expansion in subgenus *Idiophyton*. Therefore, a taxonomic re‐evaluation of subgeneric classification incorporating these genomic structural features together with traditional morphological characters is warranted.

*Lysimachia thyrsiflora* , previously classified within subgenus *Naumburgia*, was recovered as forming a monophyletic clade with members of subgenus *Lysimachia* section *Lysimachia*, including *L. davurica* and *L. vulgaris* (Feng‐Hwai and Chi‐Ming 1979). This plastome‐based relationship supports a close relationship between *L. thyrsiflora* and members of section *Lysimachia* and provides chloroplast genomic evidence relevant to evaluating its phylogenetic placement.

Unlike *L. davurica* and *L. vulgaris* , which are distributed in Eurasia, *L. thyrsiflora* has a disjunct Eurasian–North American distribution. This pattern may reflect a complex biogeographic history involving historical range expansion, extinction, or long‐distance dispersal. The Bering Land Bridge has been proposed as a potential migration corridor for intercontinental plant exchange between East Asia and North America during the Neogene (Jiang et al. 2019). Given previous hypotheses that *Lysimachia* originated in East Asia (Yan et al. 2018; Deng et al. 2020), a Beringian migration scenario represents one plausible explanation for the present distribution of *L. thyrsiflora* .

However, this interpretation should be regarded as tentative because the present study did not conduct ancestral‐area estimation or model‐based biogeographic reconstruction. Although a time‐calibrated plastome phylogeny was inferred, the present data do not establish the ancestral area, dispersal direction, timing, or route of *L. thyrsiflora* . Future studies incorporating broader sampling across *Lysimachia*, population‐level sampling of *L. thyrsiflora* across its Eurasian and North American range, nuclear genomic data, geographic occurrence data, and formal biogeographic models will be necessary to test alternative scenarios, including Beringian migration.

In contrast, *L. coreana* , a species endemic to Korea and currently treated as a synonym of *L*. *davurica* in Kew's database, formed an independent clade, consistent with previous molecular studies (Liu et al. 2023; National Institute of Biological Resources 2021). Despite its morphological similarity to other section *Lysimachia* species, the distinct phylogenetic placement of *L. coreana* reinforces its status as a genetically unique lineage, despite its current treatment as a synonym, and highlights the need for taxonomic re‐evaluation.

The divergence time analysis indicated that the genus *Lysimachia* diverged in the early Oligocene, approximately 33 Mya, coinciding with the Eocene–Oligocene transition, a period marked by temperature decline and vegetation turnover (Figure 7) (Prothero 1994; Flower and Kennett 1994). Most subgenera began to diverge around 15 Mya, and following the Middle Miocene Climate Optimum and subsequent global temperature decline, many *Lysimachia* species underwent diversification (Zachos et al. 2001; Rieseberg and Soltis 1991). These findings suggest that two major cooling events influenced the speciation within *Lysimachia*, suggesting the significant role of climatic changes in shaping the diversification of the genus. However, because this inference is based solely on chloroplast genome data, it may not fully capture the evolutionary history of the genus. Chloroplast genomes are typically maternally inherited. Therefore, plastome‐based phylogenetic analyses may not always reflect species‐level evolutionary relationships, as processes such as hybridization, incomplete lineage sorting, and chloroplast capture can lead to discordance between plastome and nuclear phylogenies (Rieseberg and Soltis 1991; Folk et al. 2017). However, because this inference is based solely on chloroplast genome data, it may not fully capture the complex evolutionary history of *Lysimachia*. Chloroplast genomes represent a single, typically maternally inherited genomic compartment and may be influenced by evolutionary processes such as incomplete lineage sorting, hybridization, and chloroplast capture, which can result in discordance between plastid and nuclear phylogenies. Therefore, transcriptome‐based phylogenomic analyses incorporating nuclear genomic data are needed to further evaluate the phylogenetic relationships and divergence patterns within *Lysimachia* and to provide a more comprehensive understanding of the evolutionary history of the genus. Furthermore, the non‐monophyly observed in several subgenera and sections warrants further investigation using independent nuclear genomic evidence and may provide a basis for future taxonomic re‐evaluation.

## 5 Conclusion

In this study, we sequenced and characterized the cp genomes of four *Lysimachia* species, providing essential genomic data for taxonomic resolution. Comparative analysis identified hot‐spot regions (*ycf*1, *ndh*F‐*rpl*32) that serve as potential molecular markers. Analysis of substitution rates identified elevated *Ka*/*Ks* ratios in the *rps*12 and *psa*I genes, providing preliminary signals that require further validation using codon‐based selection tests. Phylogenetic analysis indicated that the genus *Lysimachia* forms a monophyletic group with a high bootstrap value. In addition, *L. thyrsiflora* was found to form a monophyletic group with subgenus *Lysimachia* section *Lysimachia* rather than its traditional classification. Finally, divergence time analysis suggested that the genus *Lysimachia* diversified during the early Oligocene (33 Mya). In conclusion, we clarified the phylogenetic positions of taxa within the genus *Lysimachia* and identified potential molecular markers. Moreover, the results suggest that a taxonomic reassessment of subgenera based on molecular phylogenetic evidence may be necessary.

## Author Contributions

**SeongJun Park:** conceptualization (equal), data curation (lead), formal analysis (lead), investigation (lead), methodology (lead), resources (equal), validation (equal), visualization (equal), writing – original draft (lead), writing – review and editing (equal). **JeongYun Byeon:** data curation (equal), methodology (equal), validation (equal), visualization (equal). **SeonJoo Park:** conceptualization (equal), funding acquisition (lead), project administration (lead), resources (equal), validation (equal), writing – review and editing (equal).

## Funding

This research was supported by the 2026 Yeungnam University research grant and Basic Science Research Program Through the National Research Foundation of Korea (NRF) funded by the Ministry of Education (RS‐2025‐25407375).

## Conflicts of Interest

The authors declare no conflicts of interest.

## Supporting information


**Figure S1:** cp genome sequence alignment of 49 *Lysimachia* species with *L. europaea* serving as a reference. Sequence similarities were calculated and visualized using mVISTA.

**Figure S2:** Comparisons of junctions among the cp genomes of *Lysimachia*. JLB, the junction between the LSC/IRb; JSB, the junction between the SSC/IRb; JSA, the junction between the SSC/IRa; JLA, the junction between the LSC/IRa.

**Figure S3:** Divergence time estimation of *Lysimachia* species based on 76 chloroplast protein‐coding genes from 49 *Lysimachia* species and three outgroup taxa. Blue boxes indicates the 95% highest posterior density (HPD) intervals on the chronogram.

**Table S1:** List of taxa and GenBank accession numbers used in the study.

**Table S2:** List of genes present in the chloroplast genomes of four *Lysimachia* species.

**Table S3:** Distribution of distinct types of repeats in the 49 Lysimachia cp genomes.

**Table S4:** Amount of synonymous and non‐synonymous substitution rates present in the 76 protein‐coding genes of the 49 Lysimachia cp genomes.

## Data Availability

All plastomes generated in this study have been deposited in GenBank at the National Center for Biotechnology Information (NCBI): *Lysimachia acroadenia* (PQ783062), *L. leucantha* (PQ784106), *L. thyrsiflora* (PQ783063), and *L. arvensis* (PQ774309) (Table S1). The data that support the findings of this study are available in the Supporting Information of this article.
