# Supplementary material for: Plastome‐Based Phylogenomics Resolves Phylogenetic Complexity in Lysimachia and Reveals Unexpected Placement of L. thyrsiflora
Source: Ecol Evol. 2026 Nov 4;16(11):e74544. doi: 10.1002/ece3.74544 (PMC13734742; doi:10.1002/ece3.74544)
Supplement: Supplementary file 1 [file ECE3-16-e74544-s001.docx]

**Table S2.** List of genes present in the chloroplast genomes of four *Lysimachia* species.

| Category of Genes | Group of Genes | Gene Name |
| --- | --- | --- |
| Photosynthesis-related genes | ATP synthase | *atp*A, *atp*B, *atp*E, *atp*F*, *atp*H, *atp*I |
|  | Large subunit of rubisco | *rbc*L |
|  | Photosystem Ⅰ | *psa*A, *psa*B, *psa*C, *psa*I, *psa*J |
|  | Assembly / stability of photosystem Ⅰ | y*cf*3**, *ycf*4 |
|  | Photosystem Ⅱ | *psb*A, *psb*B, *psb*C, *psb*D, *psb*E, *psb*F, *psb*H, *psb*I, *psb*J, *psb*K, *psb*L, *psb*M, *psb*N, *psb*T, *psbZ* |
|  | Cytochrome b6/f complex | *pet*A, *pet*B*, *pet*D*, *pet*G, *pet*L, *pet*N |
|  | Cytochrome c synthesis | *ccs*A |
|  | NADH dehydrogenase | *ndh*A*, *ndh*B*(*2), *ndh*C, *ndh*D, *ndh*E, *ndh*F, *ndh*G, *ndh*H, *ndh*I, *ndh*J, *ndh*K |
| Transcription and translation related genes | RNA polymerase subunits / transcription | *rpo*A, *rpo*B, *rpo*C1*, *rpo*C2 |
|  | Small subunit of ribosomal proteins | *rps*2, *rps*3, *rps*4, *rps*7(*2), *rps*8, *rps*11, *rps*12**(*2), *rps*14, *rps*15, *rps*16*, *rps*18, *rps*19 |
|  | Large subunit of ribosomal proteins | *rpl*2*(*2), *rpl*14, *rpl*16*, *rpl*20, *rpl*22, *rpl*23(*2), *rpl32, rpl*33, *rpl*36 |
|  | Translation initiation factor | *inf*A |
| RNA genes | Ribosomal RNA | *rrn*4.5(*2), *rrn*5(*2), *rrn*16(*2), *rrn*23(*2) |
|  | Transfer RNA | *trn*A-UGC*(*2), *trn*C-GCA, *trn*D-GUC, *trn*E-UUC, *trn*F-GAA, *trn*G-GCC, *trn*G-UCC*, *trn*H-GUG, *trn*I-GAU*(*2), *trn*K-UUU*, *trn*L-CAA(*2), *trn*L-UAA*, *trn*L-UAG, *trnf*M-CAU, *trn*M-CAU(*2), *trn*N-GUU(*2), *trn*P-UGG, *trn*Q-UUG, *trn*R-ACG(*2), *trn*R-UCU, *trn*S-GCU, *trn*S-GGA, *trn*S-UGA, *trn*T-GGU, *trn*T-UGU, *trn*V-GAC(*2), *trn*V-UAC*, *trn*W-CCA, *trn*Y-GUA |
| Other genes | RNA processing | *mat*K |
|  | Carbon metabolism | *cem*A |
|  | Fatty acid synthesis | *acc*D |
|  | Proteolysis | *clp*P** |
|  | Component of TIC complex | *ycf*1 |
|  | Hypothetical proteins | *ycf*2(*2) |

* Gene with one intron, ** Gene with two introns, (*2) Gene with two copies.
