# Supplementary material for: Plastome‐Based Phylogenomics Resolves Phylogenetic Complexity in Lysimachia and Reveals Unexpected Placement of L. thyrsiflora
Source: Ecol Evol. 2026 Nov 4;16(11):e74544. doi: 10.1002/ece3.74544 (PMC13734742; doi:10.1002/ece3.74544)
Supplement: Supplementary file 2 [file ECE3-16-e74544-s002.docx]

**Table S1.** List of taxa and GenBank accession numbers used in the study.

| S. N. | Species name | NCBI number |
| --- | --- | --- |
| 1. | *Lysimachia acroadenia* | PQ783062 (In this study) |
| 2. | *Lysimachia alfredii* | LC758783 |
| 3. | *Lysimachia arvensis* | PQ784106 (In this study) |
| 4. | *Lysimachia auriculata* | LC758791 |
| 5. | *Lysimachia barystachys* | LC758794 |
| 6. | *Lysimachia candida* | NC_063330 |
| 7. | *Lysimachia capillipes* | LC758781 |
| 8. | *Lysimachia chekiangensis* | LC758807 |
| 9. | *Lysimachia christiae* | NC_053736 |
| 10. | *Lysimachia circaeoides* | LC758808 |
| 11. | *Lysimachia clethroides* | NC_064345 |
| 12. | *Lysimachia congestiflora* | NC_045275 |
| 13. | *Lysimachia coreana* | NC_026197 |
| 14. | *Lysimachia davurica* | LC758805 |
| 15. | *Lysimachia decurrens* | LC758785 |
| 16. | *Lysimachia deltoidei* var. *cinerascens* | LC758792 |
| 17. | *Lysimachia europaea* | LC758812 |
| 18. | *Lysimachia fistulosa* var. *wulingensis* | LC758786 |
| 19. | *Lysimachia foenum-graecum* | LC758788 |
| 20. | *Lysimachia fordiana* | NC_065763 |
| 21. | *Lysimachia fortunei* | LC758799 |
| 22. | *Lysimachia fukienensis* | LC758787 |
| 23. | *Lysimachia glanduliflora* | LC758778 |
| 24. | *Lysimachia grammica* | LC758800 |
| 25. | *Lysimachia hemsleyana* | LC758804 |
| 26. | *Lysimachia hemsleyi* | LC758779 |
| 27. | *Lysimachia heterogenea* | LC758773 |
| 28. | *Lysimachia hypericoides* | LC758790 |
| 29. | *Lysimachia insignis* | LC758772 |
| 30. | *Lysimachia japonica* | LC758796 |
| 31. | *Lysimachia klattiana* | LC758774 |
| 32. | *Lysimachia laxa* | LC758780 |
| 33. | *Lysimachia leucantha* | PQ783063 (In this study) |
| 34. | *Lysimachia lobelioides* | LC758793 |
| 35. | *Lysimachia longipes* | LC758806 |
| 36. | *Lysimachia maritia* | MW029425 |
| 37. | *Lysimachia mauritiana* | NC_060700 |
| 38. | *Lysimachia melampyroides* | LC758810 |
| 39. | *Lysimachia microcarpa* | LC758776 |
| 40. | *Lysimachia paridiformis* var. *stenophylla* | LC758802 |
| 41. | *Lysimachia parvifolia* | LC758775 |
| 42. | *Lysimachia patungensis* | LC758797 |
| 43. | *Lysimachia pentapetala* | LC758801 |
| 44. | *Lysimachia phyllocephala* | LC758803 |
| 45. | *Lysimachia pseudohenryi* | LC758777 |
| 46. | *Lysimachia rubiginosa* | LC758811 |
| 47. | *Lysimachia stenosepala* | LC758809 |
| 48. | *Lysimachia thyrsiflora* | PQ774309 (In this study) |
| 49. | *Lysimachia vulgaris* | LC758795 |
| 50. | *Cyclamen graecum* | OP957067 |
| 51. | *Cyclamen hederifolium* | ON480523 |
| 52. | *Cyclamen persicum* | OP526391 |
